# The American System of Dentistry

**Published:** 1887-10

**Authors:** 


### Bibliographical.

The American System of Dentistry.—In treatises by various authors. Edited by Wilbur F. Litch, M. D., D. D. S., Professor of Prosthetic Dentistry, Therapeutics and Materia Medica in the Pennsylvania College of Dental Surgery.

The third volume of this voluminous work has been issued, and compares most favorably with the preceding volumes.

The contents of volume III consist of seven parts which treat of the following subjects: Part I. Anæsthesia and Anæsthetics, by Wilbur F. Litch, M. D., D. D. S. Part II. Physiology of Digestion, by A. S. Brubaker, A. M., M. D. Physiology of Voice and Speech, by Carl Seiler, M. D. Part III. Diseases Incident to First Dentition, by James W. White, M. D., D. D. S. The Causes of Congenital Defectiveness and Deformity of the Teeth, by A. H. Thompson, D. D. S. Anomalies of the Teeth and Maxillæ, by S. H. Guilford, A. M., D. D. S. Hypercementosis, by S. H. Guilford, A.M., D. D. S. Reflex Neurosis Associated with Dental Pathology, by A. P. Brubaker, A. M., D. D. S. Inflammation of the Mucous Membrane of the Oral, Nasal and Pharyngeal Cavities, by W. T. Sudduth, M. D., D. D. S. Oral Surgery, Part I, by L. McLane Tiffany, M. A., M. D. Oral Surgery, Part II, by John H. Packard, A. M., M. D. Part IV. The Eruption and Structural Relations of the Deciduous and Permanent Teeth, by C. N. Pierce, D. D. S. Part V. Materia Medica and Therapeutics, by Henry Leffman, M. D., D. D. S. Part VI. Dental Metallurgy, by Ed C Kirk, D. D. S. Dental Jurisprudence, by Chas. G. Garrison, M. D. All constituting a valuable addition to dental literature. Publishers: Lea Brothers & Co., Philadelphia, 1887.

